# Prevalence of suicidal thoughts and attempts in the transgender population of the world: a systematic review and meta-analysis

**DOI:** 10.1186/s12991-023-00460-3

**Published:** 2023-08-05

**Authors:** Parisa Kohnepoushi, Maziar Nikouei, Mojtaba Cheraghi, Parsa Hasanabadi, Hamza Rahmani, Maryam Moradi, Ghobad Moradi, Farhad Moradpour, Yousef Moradi

**Affiliations:** 1 Student Research Committee, Kurdistan University of Medical Sciences, Sanandaj, Iran; 2 Social Determinants of Health Research Center, Research Institute for Health Development, Kurdistan University of Medical Sciences, Sanandaj, Iran

**Keywords:** Suicidal thoughts, Suicidal attempts, Transgender, Meta-analysis

## Abstract

**Background:**

The aim of this meta-analysis was to determine global pooled prevalence of suicide thoughts and attempts in transgender population.

**Methods:**

For doing comprehensive search strategy related to objectives in the presence meta-analysis, all international databases like PubMed (Medline), Scopus, Embase, Web of Sciences, PsycINFO, and the Cumulative Index to Nursing and Allied Health Literature (CINHAL) were searched from January 1990 to December 2022. The quality of the final selected studies was evaluated according to Newcastle–Ottawa Quality Assessment Scale for cross-sectional studies. The subgroup analysis was done based on type of transgender (female to male, male to female) and prevalence (point, period, and lifetime), country, and criteria of diagnosis. All analysis was done in STATA version 17.

**Results:**

From the total number of 65 selected studies, 71 prevalence of suicidal thoughts, including point, period, and lifetime prevalence were extracted and combined. After combining these values, the prevalence of suicidal thoughts in the transgender population in the world was 39% in the past month (pooled point prevalence: 39%; 95% CI 35–43%), 45% in the past year (pooled period prevalence: 45%; % 95 CI 35–54%) and 50% during lifetime (pooled lifetime prevalence: 50%; % 95 CI 42–57%). Also, the prevalence of suicide attempt in the transgender population of the world was 16% in the past month (pooled point prevalence: 16%; 95% CI 13–19%), 11% in the past year (pooled period prevalence: 11%; % 95 CI 5–19%) and 29% during lifetime (pooled lifetime prevalence: 29%; % 95 CI 25–34%).

**Conclusion:**

The present meta-analysis results showed the prevalence of suicidal thoughts and attempts in the transgender community was high, and more importantly, about 50% of transgenders who had suicidal thoughts, committed suicide.

**Supplementary Information:**

The online version contains supplementary material available at 10.1186/s12991-023-00460-3.

## Background

Transgender is a comprehensive term which includes the definitions of transsexuals, trans people, travesties, another gender identity and people whose gender identity or gender expression is different from their gender at birth. There are two groups of transsexuals including trans-women groups (male to female/MTF) referring to people whose gender was male at the time of birth but they consider themselves as women and trans-men groups (female to male/FTM) referring to people who are women at the time of birth but they accept themselves as men and are gender-nonconforming individuals [1, 2]. Transgenders are a minority group of society but their population is under growing and needs focusing on their life problems and life-threatening factors [3, 4].

In groups excluded from society, including the Black population, refugees, immigrants, lesbians, homosexuals, bisexuals, transgenders, intersex people and indigenous people, a higher prevalence of negative mental health outcomes such as depression, anxiety, substance use disorders, suicide attempts and suicidal thoughts has been reported. Suicide is one of the major problems in the transgender community. According to statistics of the World Health Organization (WHO), the annual global prevalence of suicide has increased and become a public health problem while it is the leading cause of death in the United States (US) and the 10^th^ leading cause of death among youth and adolescents [5]. Effective prevention measures include identifying people at risk, understanding the circumstances of suicide and evaluating the effectiveness of interventions [2]. It has been estimated that 5.6–14.4% of all people in society think about suicide at some point in their lives and 1.9–8.7% commit suicide. Furthermore, the prevalence of suicide attempts in trans people has been estimated to be 16–42% [6]. According to a longitudinal study in Sweden, the risk of death by suicide in trans people is 19 times higher than that of the control group [7]. According to a study in India, the prevalence of suicide in trans people is 32–50% [8]. Based on a study done in Argentina, the lifetime prevalence of suicide in trans-women and trans-men was reported to be 33% [1]. In a study in the US, 15% of transgender students reported committing suicide in the last 12 months and in another study in America, 41% of transgender students reported committing suicide during their lifetime [9]. One of the important factors exposing trans people to depression and suicide is prejudice towards them. In addition to the general stressful life factors, trans people are exposed to high levels of discrimination, violence and rejection due to their gender identity or its expression [2]. Among other factors related to suicide in trans people, the third one is drug use while age, mental health problems, education levels and income levels can be mentioned [1]. According to the data of the WHO, family support, friends and other important relationships, social participation, satisfactory social life and access to mental health care services are protective and preventive factors against suicide for trans people and the public [2].

In the study, we included (number of papers) and documented suicide thoughts and attempts among transgender people. The suicide was evaluated in two ways: (1) self-reporting of suicidal thoughts; (2) self-reporting of suicide attempts. The data indicated different suicide statistics in trans people and no fixed suicide statistics were available based on the regions. In this study, we calculated the suicide prevalence based on the regions and estimated the exact prevalence of suicide thoughts and attempts in trans people based on the regions.

## Methods

The study protocol has been registered on the Prospero website with the code CRD42022376073. This study was a systematic review and meta-analysis conducted with the aim of estimating the global pooled prevalence (point, period, and lifetime) of suicidal thoughts and attempts in transgender people through the following steps and based on the structure of the Preferred Reporting Items for Systematic Reviews and Meta-Analyses (Additional file 1: PRISMA 2020 Checklist) [10].

### Eligibility criteria

The study aimed to determine the global prevalence of suicidal thoughts and attempts in transgender people. All cross-sectional studies, both descriptive and analytical, were reviewed and other studies (case controls, cohorts, clinical trials, letters to the editor, case or case reports and review studies) were excluded from the study. Reporting suicidal thoughts or attempts as a frequency in cross-sectional studies and articles in English were other inclusion criteria. Also, the articles which reported the suicide (suicide thoughts or attempts) in the form of a mean score along with standard deviation and other indicators other than frequency were excluded from the study. In this way, only cross-sectional studies reporting percentage or frequency were included in the present meta-analysis. Finally, only articles conducted on the transgender population were included and ones performed on other gender minorities such as lesbians, bisexuals, gays, etc., were excluded from the study.

### Information sources and search strategy

Articles published from January 1990 to December 2022 in the international databases including Scopus, PubMed (Medline), Web of Science, PsycoInfo, the Cumulative Index to Nursing and Allied Health Literature (CINHAL), and Embase were retrieved and screened. The main keywords in this study included "Suicide Attempt", "Suicide Ideation", and "Transgender" synonyms of the main keywords in the Mesh search engine. In addition, by examining other studies, other synonyms were also found and used in the search strategy. In order to carry out a comprehensive search strategy, the authors also searched the first ten pages of google scholar and selected related articles. In addition, manual search was also performed by checking the resources of related articles. After creating a library in Endnote software version 9 for each database, they were placed in a combined form and duplicate articles were removed based on the software default. Then, the remaining articles were evaluated based on their title, abstract and full text considering the inclusion criteria. Two of the authors (PK and MN) independently and separately screened the articles based on their title, abstract and full text and if there was a discrepancy, the results were reviewed by the supervisor.

### Data extraction

After screening the articles to identify those that were relevant to the study’s purpose, a checklist was created based on the opinions of experts. This checklist included several components, such as the authors’ names, study type, publication year, total sample size, country, continent, population type, age, and frequency of suicidal thoughts or attempts. The purpose of using this checklist was to ensure that all relevant data were extracted from each selected studies in this meta-analysis and consistent manner. By using a standardized checklist, the researchers could reduce the risk of bias and increase the reliability of the data extracted from each study.

### Risk of bias

NOS (Newcastle–Ottawa Quality Assessment Scale) checklist was used to evaluate the risk of bias [11]. This checklist has been designed to evaluate the quality of cross-sectional studies. Each of the items is given a score of 1 if observed in the studies. The maximum score for each study is 9. This step was independently performed by two authors (PK and MCH) and in case of disagreement, the cases were referred to the third researcher (YM).

### Statistical analysis

To perform the analysis, the total sample size of the studies along with the number of transgender people with suicidal thoughts or attempts were extracted from all the selected studies for meta-analysis. According to the extracted information, the Metaprop command was used to calculate the pooled estimate and the results were analyzed. To check the heterogeneity and variance between the selected studies, Cochran’s *Q* and *I*2 tests were used. Statistical analysis was performed using STATA 17 and P-value < 0.05 was considered. In addition, funnel plot and Egger’s test were used to check publication bias. Meta-regression analysis was also applied to investigate the effect of age on the prevalence of suicidal thoughts and attempts. Finally, subgroup analyzes were performed and reported based on the type of gender such as FTM (female to male) or MTF (male to female), the continent and type of prevalence (point, period and lifetime) which point prevalence indicates the prevalence in the past month, period prevalence indicates the prevalence in the past year and life time prevalence indicates the prevalence during the lifetime.

## Results

### Qualitative results

**Fig. 1:**
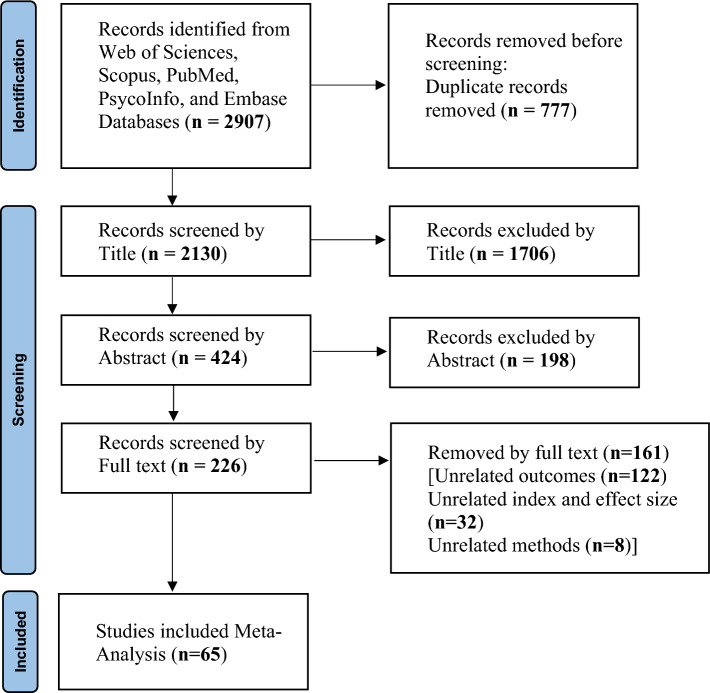
A flow diagram demonstrating the study selection process

In this meta-analysis, after completing the search in the desired databases, 2907 studies were retrieved. After removing duplicates (777 studies), 2130 were screened based on their titles and 1706 studies were excluded while 424 ones were entered into the screening stage based on the abstract. In the screening stage based on the abstract, 198 studies were excluded and 226 were included in the screening stage based on the full text. Finally, at this stage, 161 studies were excluded and 65 related studies were selected and entered into the meta-analysis (Fig. 1).

Data of the 65 selected studies are summarized in Table 1. The community investigated in these studies was different types of the transgender community such as FTM and MTF. All studies were descriptive or analytical cross-sectional, conducted in different countries and diverse societies. From the 65 selected studies, 21 received a scores of 6, 35 a scores of 7, and 9 a scores of 8 according to the quality assessment based on the NOS checklist (Table 1). Most of the selected studies reported the number of trans people with suicidal thoughts or attempts (Table 1).

**Table 1:** The characteristics of included studies

Author	Year	Country	Study design	Study population	Mean age	Sample size	Suicidal ideation	Suicide attempt	NOS score
Andreas K	2021	European countries	Cross-sectional	TS	30.7	5267	1827	1797	7
Yossi Levi-B	2022	Israel	Cross-sectional	TS	55	195	151	77	6
Shira M	2009	USA	Cross-sectional	FTM	47	153	NR	9	6
				MTF	47	153	NR	12	
Brandon D. L. M	2015	Argentina	Cross-sectional	FTM	30	482	NR	18	6
				MTF	30	482	NR	141	
Robin M. M	2002	USA	Cross-sectional	TS	36.88	73	27	17	7
Azar N	2021	Iran	Cross-sectional	MTF	27.6	127	91	64	7
Arianne R	2021	Brazil	Cross-sectional	MTF	30	763	191	238	7
Sari L. R	2014	USA	Cross-sectional	TS	37.3	31	18	9	7
Katharine A.R	2019	UK	Cross-sectional	FTM	19.7	210	108	46	7
				MTF	20.2	105	58	24	
Ankur S	2021	California	Cross-sectional	TS	NR	120	NR	17	6
				VTS	NR	120	NR	51	
Gareth J. T	2020	Australia	Cross-sectional	TS	26	392	157	66	6
				VTS	26	392	367	209	
Raymond P.T	2017	USA	Cross-sectional	TS	NR	170	62	NR	6
					NR	170	99	NR	
					41.14	36	14	NR	
					41.14	36	19	NR	
Jack L. T	2020	USA	Cross-sectional	TS	23.4	3494	3129	1775	8
Sahika Y	2015	Turkey	Cross-sectional	MTF	NR	42	23	13	7
				FTM	NR	99	55	29	
Galit Z	2018	Sweden	Cross-sectional	MTF	NR	149	60	54	8
				FTM	NR	187	75	79	
				TS	NR	796	283	251	
Sav Z	2021	Australia	Cross-sectional	TS	28	928	NR	394	8
Ashley A	2020	USA	Cross-sectional	TS	NR	372	320	208	7
Greta R. B	2015	Canada	Cross-sectional	TS	32.7	380	133	43	7
Runsen C	2019	China	Cross-sectional	MTF	23.3	687	413	141	7
				FTM	23.3	622	319	68	
Itala Raymundo C	2021	Brazil	Cross-sectional	TS	26.8	378	256	163	7
Ricardo de M. R. R	2021	Brazil	Cross-sectional	MTF	NR	345	163	64	6
Anthony F	2020	USA	Cross-sectional	TS	NR	572	85	184	6
				VTS	NR	572	40	184	
Peter G	2012	USA	Cross-sectional	TS	37.1	290	NR	83	6
Arnold H. G	2016	USA	Cross-sectional	TS	18	129	52	29	6
Shelley H	2021	USA	Cross-sectional	TS	22.5	3471	977	179	6
		Canada	Cross-sectional	TS	22.5	717	196	54	
Adam G. H	2020	USA	Cross-sectional	MTF	NR	33	10	8	7
				FTM	NR	98	45	30	
Sanna S	2021	Nepal	Cross-sectional	MTF	NR	139	58	26	7
Yiu T. S	2017	Hong Kong	Cross-sectional	TS	NR	106	71	22	6
Seishi T	2011	Japan	Cross-sectional	MTF	32.2	188	141	NR	6
				FTM	26.4	312	219	NR	
Brian C. T	2019	USA	Cross-sectional	TS	NR	1148	974	577	7
Galit Z	2018	Sweden	Cross-sectional	MTF	NR	149	60	54	7
				FTM	NR	187	75	79	
Sav Z	2021	Australia	Cross-sectional	TS	NR	928	NR	399	7
Rao A	2019	Pakistan	Cross-sectional	TS	39	156	67	NR	6
Itala R. C	2019	Brazil	Cross-sectional	TS	26.8	378	256	163	6
Collier M. C	1997	USA	Cross-sectional	MTF	32	318	38	NR	7
				FTM	30	117	25	NR	
Rachel L	2016	Lebanon	Cross-sectional	TS	27	54	NR	25	7
Aboussouan A	2019	USA	Cross-sectional	TS	49.01	154	NR	110	7
				TS	49.01	159	NR	56	
				TS	31.11	2841	NR	1540	
				TS	31.11	1131	NR	226	
				TS	49.01	154	98	21	
				TS	49.01	159	43	6	
				TS	31.11	2841	1936	392	
				TS	31.11	1131	432	40	
Yockey A	2020	USA	Cross-sectional	TS	NR	790	272	287	7
Azeem R	2019	Pakistan	Cross-sectional	TS	39.26	156	67	NR	6
Bauer G	2015	Canada	Cross-sectional	TS	32.7	380	NR	43	7
Boyer L	2021	USA	Cross-sectional	TS	NR	8112	NR	66	7
Chang R	2022	China	Cross-sectional	MTF	34	198	73	NR	7
Chen R	2019	China	Cross-sectional	MTF	22.89	687	413	141	7
				FTM	23.78	622	319	68	
				TS	23.31	1309	732	209	
Chen Y	2020	China	Cross-sectional	MTF	27.9	250	55	64	7
Chinazzo I	2021	Brazil	Cross-sectional	TS	NR	378	256	163	7
Clements-N. K	2006	USA	Cross-sectional	MTF	NR	392	NR	127	7
				FTM	NR	123	NR	39	
Drakeford L	2018	USA	Cross-sectional	TS	NR	6450	NR	3394	8
Duffy M	2018	USA	Cross-sectional	TS	NR	122	92	91	7
				TS	NR	436	102	33	
Green A	2021	USA	Cross-sectional	TS	19.95	1216	NR	173	7
				TS	16.91	4537	NR	956	
Grossman A	2007	USA	Cross-sectional	MTF	17.5	31	12	6	7
				FTM	19.5	24	13	8	
Jin H	2020	USA	Cross-sectional	MTF	23.2	297	56	NR	7
Kittiteerasack P	2020	Thailand	Cross-sectional	TS	NR	96	22	NR	8
				TS	NR	96	39	12	
Kota K	2020	USA	Cross-sectional	MTF	35	92	30	NR	7
Kuper L	2018	USA	Cross-sectional	TS	21.07	1896	1587	207	7
Lehavot K	2016	USA	Cross-sectional	TS	49.28	212	120	68	7
Maksut J	2020	USA	Cross-sectional	MTF	NR	381	226	50	8
Marshall B	2015	Argentina	Cross-sectional	TS	30	482	NR	159	7
Perez-Brumer A	2017	USA	Cross-sectional	TS	15.23	7653	2467	NR	7
Perez-Brumer A	2015	USA	Cross-sectional	TS	32.74	1229	NR	355	8
				TS	32.74	1229	NR	51	
Peterson C	2016	USA	Cross-sectional	TS	17.1	96	NR	27	6
Rabasco A	2020	USA	Cross-sectional	TS	26.44	133	NR	62	6
Rabasco A	2021	USA	Cross-sectional	TS	26.01	180	NR	75	6
Schweizer V	2020	USA	Cross-sectional	MTF	NR	229	131	NR	7
				FTM	NR	121	94	NR	
Seelman L	2016	USA	Cross-sectional	TS	31.02	2325	NR	1081	6
Mustanski B	2010	USA	Cross-sectional	TS	NR	20	2	2	6

MTF: male to female; FTM: female to male; TS: transgender; VTS: veteran transgender

## Quantities results

### Suicidal thoughts in the transgender

From the total number of 65 selected studies, 71 prevalence of suicidal thoughts, including point, period, and lifetime prevalence were extracted and combined. After combining these values, the meta-analysis results showed the pooled prevalence of suicidal thoughts in the transgender community of the whole world was equal to 48% (pooled prevalence: 48%; 95% CI 42–54%; *I*_square_: 88.53%; *P*_heterogeneity_ < 0.0001). In the present meta-analysis, the overall prevalence was also calculated into three states of point, period, and lifetime prevalence and the results showed these values were equal to 39%, 45%, and 50%, respectively, in the transgender community of the world. In this way, the prevalence of suicidal thoughts in the transgender community in the world was 39% in the past month (pooled point prevalence: 39%; 95% CI: 35–43%), 45% in the past year (pooled period prevalence: 45%; % 95 CI 35–54%) and 50% during lifetime (pooled lifetime prevalence: 50%; % 95 CI 42–57%) (Table 2).

**Table 2:** The pooled prevalence of suicide ideation in transgender population based on type of TS, and continents

Outcomes	Number of studies	Pooled prevalence %	95% CI		Heterogeneity assessment		
			Lower %	Upper %	*I* square %	Z	*P* value
Overall prevalence	71	48	42	54	88.53	26.62	< 0.0001
Type of prevalence							
Point prevalence	3	39	35	43	86.99	22.09	< 0.0001
Period prevalence	16	45	35	54	89.93	26.33	< 0.0001
Life time prevalence	52	50	42	57	88.43	26.41	< 0.0001
Type of TS							
Female to male							
Overall	11	51	43	59	93.40	12.28	< 0.0001
Period prevalence	2	40	35	45	93.71	12.22	< 0.0001
Life time prevalence	9	53	44	62	88.90	10.33	< 0.0001
Male to female							
Overall	20	44	35	53	97.56	19.21	< 0.0001
Period prevalence	3	47	33	61	80.09	16.00	< 0.0001
Life time prevalence	17	43	33	53	90.88	18.29	< 0.0001
Both							
Overall	40	49	41	58	89.60	17.05	< 0.0001
Period prevalence	11	45	33	57	89.68	11.45	< 0.0001
Point prevalence	3	39	35	43	98.88	30.36	< 0.0001
Life time prevalence	26	53	40	65	94.66	12.45	< 0.0001
Continents							
Europe							
Overall	10	43	39	48	86.44	27.69	< 0.0001
Period prevalence	5	38	35	40	0.00	47.97	0.500
Life time prevalence	5	50	38	62	93.52	11.94	< 0.0001
Asia							
Overall	17	53	46	59	95.75	23.67	< 0.0001
Period prevalence	1	23	15	33	-	8.87	< 0.0001
Life time prevalence	16	54	48	61	95.51	24.74	< 0.0001
America							
Overall	42	46	37	55	97.99	15.30	< 0.0001
Point prevalence	2	37	30	44	89.03	16.69	< 0.0001
Period prevalence	10	50	37	63	89.43	10.82	< 0.0001
Life time prevalence	30	45	33	58	88.99	11.13	< 0.0001
Australia							
Overall	2	71	68	74	88.90	54.59	< 0.0001
Continents							
Europe							
MTF	4	47	38	55	65.29	16.66	0.0380
FTM	4	46	39	54	73.46	18.85	0.0109
Both	2	35	34	37	88.00	96.18	< 0.0001
Asia							
MTF	7	52	39	65	97.26	11.68	< 0.0001
FTM	3	58	47	68	88.09	14.90	< 0.0001
Both	7	50	39	62	95.00	12.63	< 0.0001
America							
MTF	9	35	23	48	97.59	8.55	< 0.0001
FTM	4	50	22	77	96.45	4.94	< 0.0001
Both	29	49	38	60	99.68	13.06	< 0.0001
Australia							
Both	2	71	68	74	89.04	54.59	0.0089

MTF: male to female; FTM: female to male; TS: transgender; CI: confidence interval

### Subgroup analysis

The results of subgroup analyze based on the gender change type were also examined. In total, 11 studies reported the prevalence of suicidal thoughts in the FTM trans community and 20 studies reported it in the MTF trans community. The rest of the selected studies did not report suicidal thoughts based on these two communities; in other words, they examined the transgender community in general to report the prevalence of suicidal thoughts (both). After combining these studies, the meta-analysis results showed the period prevalence of suicidal thoughts in the FTM community was equal to 40% (pooled period prevalence: 40%; 95% CI 35–45%) while the lifetime prevalence in this community was equal to 53% (pooled lifetime prevalence: 53%; 95% CI 44–62%). The overall prevalence of suicidal thoughts in this trans community was equal to 51% (pooled prevalence: 51%; 95% CI 43–59%) whereas in the MTF community, this prevalence was equal to 44% (pooled prevalence: 44%; 95% CI 35–53%). In the MTF community, the period prevalence and lifetime prevalence were equal to 47% (pooled period prevalence: 47%; 95% CI 33–61%) and 43% (pooled lifetime prevalence: 43%; 95 CI 33–53%), respectively (Table 2).

The prevalence of suicidal thoughts among transgender people in the world was different based on the different continents so that the present meta-analysis showed in America, the period prevalence of suicidal thoughts in the transgender community was equal to 50% (pooled period prevalence: 50%; 95% CI 37–63%) while this prevalence in European and Asian transgenders was 38% (pooled period prevalence: 38%; 95% CI 35–40%) and 23% (pooled period prevalence: 23%; 95% CI 33–15%), respectively. However, the lifetime prevalence in the transgender community living in the Asian continent was equal to 54% (pooled lifetime prevalence: 54%; 95% CI 48–61%) which was more than that of transgenders living in other continents such as Europe and America (Table 2).

The prevalence of suicidal thoughts in the different continents based on FTM and MTF gender change was also investigated and compared, the results of which are shown in Table 2. The results of the meta-analysis showed the prevalence of suicidal thoughts in the MTF community of Europe was equal to 47% (pooled prevalence: 47%; 95% CI 38–55%), in America, it was equal to 35% (pooled prevalence: 35%; 95% CI 23—48%) and in Asia, it was equal to 52% (pooled prevalence: 52%; 95% CI 65–39%). While the prevalence of suicidal thoughts in the European FTM community was equal to 46% (pooled prevalence: 46%; 95% CI 39–54%), in America, it was equal to 50% (pooled prevalence: 50%; 95% CI 22–77%) and in Asia, it was equal to 58% (pooled prevalence: 58%; 95% CI 68–47%). In general, the meta-analysis results showed the prevalence of suicidal thoughts in the trans community living in Asia was equal to 53% (pooled prevalence: 53%; 95% CI 46–59%) which was more than those of Europe and America (Table 2).

### Publication bias and meta-regression

**Fig. 2:**
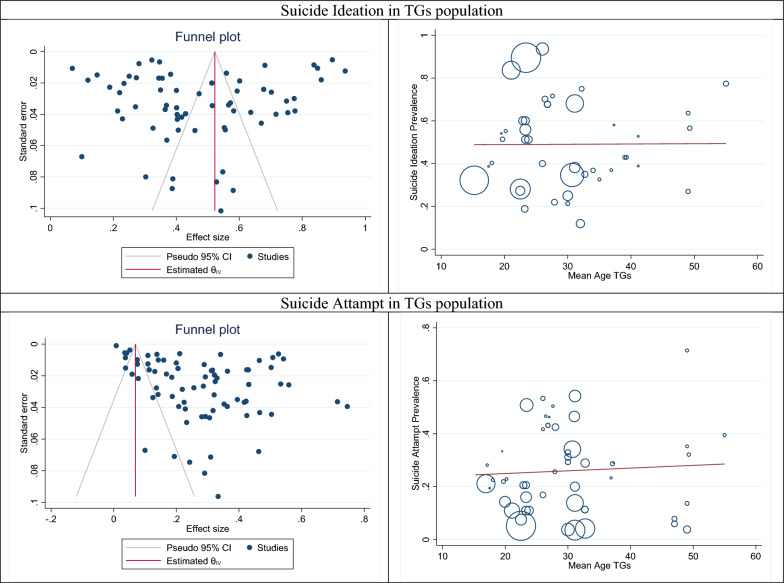
The funnel and meta-regression plots related to pooled prevalence suicide in transgender

The results of publication bias and meta-regression based on the transgender population’s age are shown in Fig. 2. According to the results of this figure and Eggers' test (B: − 0.38; SE: 0.174; P: 0.0477), publication bias has occurred in these results. Trim and fill analysis was used to more closely examine the publication bias effect on the estimated pooled prevalence, the results of which also showed it did not significantly affect the overall result (Fig. 2). The meta-regression results showed with age, the prevalence of suicidal thoughts in the transgender community slightly increased but it was not statistically significant (B: 0.0001; SE: 0.0033; P: 0.969) (Fig. 2).

### Suicidal attempts in the transgender

From the total number of 65 selected studies, 82 prevalence of suicidal attempts, including point, period, and lifetime prevalence were extracted and combined. The meta-analysis results showed the pooled prevalence of suicide attempts in the transgender community of the whole world was equal to 26% (pooled prevalence: 26%; 95% CI 22–31%; *I*_square_: 95.51%; *P*_heterogeneity_ < 0.0001). The overall prevalence was also calculated into three states of point, period, and lifetime prevalence and the results showed these values for suicide attempts in the transgender community of the world were equal to 16%, 11% and 29%, respectively. In this way, the prevalence of suicide in the transgender community of the world was 16% in the past month (pooled point prevalence: 16%; 95% CI 13–19%), 11% in the past year (pooled period prevalence: 11%; % 95 CI 5–19%) and 29% during lifetime (pooled lifetime prevalence: 29%; % 95 CI 25–34%) (Table 3).

**Table 3:** The pooled prevalence of suicide attempt in transgender population based on type of TS, and continents

Outcomes	Number of studies	Pooled prevalence %	95 CI		Heterogeneity assessment		
			Lower %	Upper %	*I* square %	Z	*P* value
Overall prevalence	82	26	22	31	95.51	18.01	< 0.0001
Type of prevalence							
Point prevalence	2	16	13	19	88.10	17.18	< 0.0001
Period prevalence	10	11	5	19	99.26	5.66	< 0.0001
Life time prevalence	70	29	25	34	99.11	22.54	< 0.0001
Type of TS							
Female to male							
Overall/life time prevalence	11	22	13	31	96.79	7.98	< 0.0001
Male to female							
Overall	17	25	21	30	91.14	18.83	< 0.0001
Period prevalence	1	13	10	17	–	13.54	< 0.0001
Life time prevalence	16	26	22	30	89.56	19.74	< 0.0001
Both							
Overall	54	28	22	34	98.67	14.53	< 0.0001
Period prevalence	9	11	5	20	89.31	5.29	< 0.0001
Point prevalence	2	16	13	19	88.88	17.18	< 0.0001
Life time prevalence	43	33	27	38	89.38	18.81	< 0.0001
Continents							
Europe							
Overall/life time prevalence	10	33	29	36	74.95	30.53	< 0.0001
Asia							
Overall/life time prevalence	12	23	18	28	94.51	14.17	< 0.0001
America							
Overall	56	25	19	32	99.64	13.24	< 0.0001
Point prevalence	1	14	8	22	87.33	7.56	< 0.0001
Period prevalence	10	11	5	19	89.26	5.66	< 0.0001
Life time prevalence	45	29	24	35	89.36	16.23	< 0.0001
Australia							
Overall	4	38	26	52	97.87	9.42	< 0.0001
Point prevalence	1	17	13	21	–	15.80	< 0.0001
Life time prevalence	3	46	40	52	88.79	24.57	< 0.0001
Continents							
Europe							
MTF	4	32	25	39	54.76	14.98	0.080
FTM	4	34	23	45	88.87	10.00	< 0.0001
Both	2	34	33	35	88.00	94.50	< 0.0001
Asia							
MTF	5	26	19	34	91.97	11.52	< 0.0001
FTM	2	11	9	13	88.79	22.44	< 0.0001
Both	5	26	15	38	94.41	6.94	< 0.0001
America							
MTF	8	21	15	29	93.46	10.44	< 0.0001
FTM	5	18	6	35	96.30	3.88	< 0.0001
Both	43	27	20	35	98.72	11.84	< 0.0001
Australia							
Both	4	38	26	52	97.87	9.42	< 0.0001

MTF: male to female; FTM: female to male; TS: transgender; CI: confidence interval

### Subgroup analysis

The results of subgroup analyze based on the gender change type were also examined and in total 11 studies reported the prevalence of suicide attempts in the FTM trans community in the form of lifetime prevalence. After combining these results, the pooled prevalence of suicide attempts in the FTM community was equal to 22% (pooled lifetime prevalence: 22%; 95% CI 13–31%) (Table 3).

17 studies reported the prevalence of suicide attempts in the MTF trans community. After combining these studies, the meta-analysis results showed the pooled prevalence of suicide attempts in this community was equal to 25% (pooled prevalence: 25%; 95% CI 21–30%) while the period and lifetime prevalence was equal to 13% (pooled period prevalence: 13%; 95% CI 10–17%) and 26% (pooled lifetime prevalence: 26%; 95% CI 22–34%), respectively (Table 3).

The prevalence of suicide attempts among transgender people in the world was different based on the different continents so that the present meta-analysis showed in the European continent, it was equal to 33% (pooled lifetime prevalence: 33%; 95% CI was 29–36%) while this prevalence in American and Asian transgenders was 29% (pooled lifetime prevalence: 29%; 95% CI 24–35%) and 23% (pooled lifetime prevalence: 23%; 95% CI 18–28%), respectively (Table 3).

The prevalence of suicide attempts in the different continents based on FTM and MTF gender change was also investigated and compared, the results of which are shown in Table 3. The meta-analysis results showed the prevalence of suicide attempts in the European MTF community was equal to 32% (pooled prevalence: 32%; 95% CI 25–39%), in America, it was equal to 21% (pooled prevalence: 21%; 95% CI 15–29%) and in Asia, it was equal to 26% (pooled prevalence: 26%; 95% CI 19–34%). While the prevalence of suicide attempt in the European FTM community was equal to 34% (pooled prevalence: 34%; 95% CI 23–45%), in America, it was equal to 18% (pooled prevalence: 18%; 95% CI 6–35%) and in Asia, it was equal to 11% (pooled prevalence: 11%; 95% CI 9–13%). In general, the meta-analysis results showed the prevalence of suicide attempts in the trans people living in Australia and Oceania was equal to 38% (pooled prevalence: 38%; 95% CI 26–52%) and higher than those of Europe, America and Asia (Table 3).

### Publication bias and meta-regression

The results of publication bias and meta-regression based on the transgender population’s age are shown in Fig. 2. According to the results of this figure and Egger's test (B: 2.18; SE: 0.974; *P*: 0.0214), publication bias has occurred in these results. Trim and fill analysis was used to more closely examine the publication bias effect on the estimated pooled prevalence, the results of which also showed it did not significantly affect the overall result (Fig. 2). The meta-regression results showed with increasing age, the prevalence of suicide attempts in the transgender community slightly increased but it was not statistically significant (B: 0.010; SE: 0.002; *P*: 0.666) (Fig. 2).

## Discussion

The transgender population in the world has been investigated by many different studies. Determining their exact number both in terms of prevalence and occurrence depends on the basic definition but according to a systematic review published in 2016, approximately 9.2 out of every 100,000 people in the world were transgender [12]. These results show the study of these populations and factors affecting health outcomes such as their socioeconomic status is very important. This study was a systematic review and meta-analysis conducted with the aim of accurately determining the prevalence of suicidal thoughts and attempts among transgender people in the world, in which the lifetime prevalence of suicidal thoughts and attempts in transgender people was 50% and 29%, respectively. These results prove the fact that almost half of the transgenders who have suicidal thoughts commit suicide. These results are very important for health policy makers and health decision-makers in the world, especially in the developing countries, because it shows the lack of proper efficiency and effectiveness of health, educational and therapeutic interventions to prevent suicide attempts and successful suicide in the transgender community. Various factors, including economic, social, cultural, health and medical factors play a role in increasing the prevalence of suicidal thoughts and, of course, suicide attempts.

On the other hand, the results of past studies have shown transgender people are at a higher risk of experiencing suicidal thoughts during their lifetime compared to other gender minority populations such as bisexuals and MSM (50% vs. 23% and 34%, respectively). This indicates compared to other sexual minorities, there are other factors which put transgender people at greater risk of suicidal thoughts and actions [13, 14]. Among the important influencing factors, we can mention the costs related to hormone therapy and how to access it, taking into account the space and environment of providing hormones, as well as direct and indirect costs related to it. Transgenders, whether FTM or MTF, need to receive sex hormones, especially estrogen or testosterone, for a long time and before performing any related surgery in order to establish their desired gender identity in their society, family and peers. The results of past studies have shown hormone therapy among transgender people causes beneficial effects such as strengthening psychological function and reducing depression and anxiety but it should be kept in mind that various factors have an effect on it. Some transgender people may be deprived of this type of treatment and access to it due to lack of financial ability, social stigma and discrimination, fear of gender identity disclosure and sexual and work abuse. As a result of this problem, they suffer from complications such as depression and anxiety disorders and suicidal thoughts leading to commit suicide [15–19].

In order to supply the required hormones in this society, which is considered one of their most important needs in the process of gender change, economic obstacles and problems can be considered as the most important factor in access to hormones. To solve this challenge, basically, there are two ways ahead of transgender people. First, these people have the support of their family and friends; in this case, the main challenge is the existence of stigma and discrimination in centers providing care or treatment services, such as pharmacies, hospitals, or care centers. Second, they do not have the support of their family and friends, and in other words, they have been rejected by them. In this case, they face economic problems and the existence of stigma in order to provide the needed hormones in the service centers. In the first case, transgender people, due to lack of direct access to needed hormones or other essential services, are forced to spend exorbitant economic costs to indirectly access the drugs they need. In the second case, these people are forced to find a job and create a source of income in order to cover the costs required for the provision of medicines and services. In this case, the possibility of work and sexual abuse of these people is very high. In both cases, these people are exposed to diseases and mental disorders such as depression, anxiety, seclusion and loneliness [19–24], because receiving the required hormones and necessary treatments in gender minorities especially transgender is one of the important factors in suicide prevention [25–27]. The results of past studies have shown the presence of mental disorders such as anxiety and depression in gender minorities, especially transgenders, is the main cause of suicide. Also, the results of these studies have shown due to the existence of these mental disorders, the rate of suicide in homosexuals and heterosexual people who have homosexual behavior is higher than that of heterosexual ones [28–30].

Among other worrisome cases, we can mention the high prevalence of drug use among these people so that according to reports, lifetime use of drugs such as cocaine, heroin and methamphetamines in these people was 27.1%, 26.1%, 24.9%, respectively, which can cause an increase in suicide attempts by these people. One of the risk factors exposing these people to drug use is their victimization of violence, rejection from society, family and surrounding people, and lack of access to needed facilities due to stigma and discrimination [31, 32]. On the other hand, these people are forced to have sex at a young age in order to meet their living expenses and find trust and support from the people around them. According to the results of past studies, transgender students are more likely than cisgender ones to experience the first sex before the age of 13 years and to have more than four sexual partners [31–34]. These early sexual experiences can expose these people to emotional damage caused by relationships, an increase in the risk of sexually transmitted infections and finally the occurrence of mental disorders and suicide.

Gender minorities, especially transgender people, face unique challenges and discrimination, such as high rates of institutionalized prejudice, bullying, violence, and physical attacks. Exclusion from the family, stigma and discrimination and other social factors expose transgender people to a phenomenon called internalized transphobia and a negative self-concept related to their gender identity. These feelings can include self-loathing or shame. Internalized transphobia increases the likelihood of suicide attempts in the transgender population [35–37].

Subgroup analyzes in the present meta-analysis showed the prevalence of suicidal thoughts in the trans community living in Asia was higher than that of other continents. This difference can be attributed to differences between various cultures and fixed traditions in families. In some Asian societies, such as Thailand, despite social supports, transgender people do not have a favorable acceptance in their families, which can be attributed to the existence and stability of traditional cultures in the family after several years [38]. However, the prevalence of suicide attempts in the trans community living in Australia and Oceania was higher than those of other continents. Based on the meta-analysis findings, the prevalence of suicidal thoughts in the FTM trans community in the Asian and American continents was higher than that of the MTF trans community. This may be due to the fact that in these communities, FTMs experience more victimization than MTFs and have more lifetime suicidal ideation [39]. Also, living more in stigmatizing communities can facilitate vulnerability to experiencing stigma-related stressors, which increases suicidal thoughts and attempts among FTM trans people [40]. In general, the prevalence of suicide attempts in the MTF trans community was higher than that of the MTF trans community. Previous studies have shown sexual abuse rates were higher among MTF individuals, which was significantly associated with suicide [41]. Among those known as transgender, men are consistently at higher risk of suicide than women [41]. Also, based on previous studies, trans-men report higher levels of gender discrimination compared to trans-women [42, 43].

In this study, the primary target group is transgender individuals, who are considered one of the key and high-risk populations in society. This group, along with other groups such as lesbian, gay, and bisexual, fall under the umbrella of the LGBT community (lesbian, gay, bisexual, transgender and queer people). These groups differ from each other in terms of high-risk behaviors, susceptibility to viral diseases and infections such as Hepatitis B (HBV) and C (HCV), or human immunodeficiency virus/acquired immunodeficiency syndrome (HIV/AIDS). Of course, considering their different circumstances, they also differ in terms of susceptibility to physical and mental disorders. For example, in terms of the degree of social discrimination and intolerance, these individuals face varying degrees of it, which may have an impact on the development of mental disorders and ultimately suicide. All previous studies have two comprehensive limitations. First, they have not been conducted on a specific group of LGBT individuals. This means that they have considered all of these groups as a single entity and reported the prevalence and incidence outcomes in this group as a whole, without paying attention to the differences between these groups [44–47].

The second limitation is that the published studies have mainly focused on the risk or tendency towards suicide ideation or attempts in these communities, rather than the actual prevalence and incidence of suicide. For example, a study by Mattia Marchi and colleagues [47] has specifically examined the risk of suicide attempts in LGBT communities. However, determining the exact prevalence of suicide, especially among transgender individuals, is of utmost importance for allocating healthcare services and providing necessary care in this community.

In addition, suicide can be categorized into two forms; suicide ideation and suicide attempts, and both should be investigated. However, in previous published studies, this aspect has received less attention. In the current meta-analysis, this issue has been analyzed and reported.

Other strengths of the present meta-analysis are the large number of analyzed cross-sectional studies and the subgroup analyzes based on the important variables reported in the primary studies. This can be effective in applying the present meta-analysis results to compile and allocate health services and care programs for the transgender community in different countries. In addition, the present meta-analysis results can be considered as a warning to health policy makers and health decision-makers in different countries because these results show the prevalence of suicidal thoughts and attempts in the transgender community is high and all health measures, interventions and treatment in this field should be reviewed and reformulated. On the other hand, these results for researchers and clinical researchers can open the way for more research with better ideas in the field of suicide in transgender people.

Among the limitations of the present study, we can mention the small number of studies in some countries such as Australia, which can make it possible to overestimate the overall result due to the small number of analyzed studies. Therefore, it is suggested to design and conduct survey studies with an appropriate sample size in these countries, considering all transgender groups in society in order to determine suicide prevalence.

## Conclusion

The findings of this meta-analysis highlight the high prevalence of suicidal thoughts and attempts in the transgender community, and the alarming fact that about 50% of transgender individuals who reported suicidal thoughts actually committed suicide. These results emphasize the urgent need for effective measures to prevent suicide and promote mental health in the transgender population. To address this issue, it is important to develop patient-centered and community-centered interventions that are evidence-based and culturally sensitive. Such interventions should include the implementation of social, economic, and support programs, as well as the provision of necessary counseling and mental health services to both transgender individuals and their families. In addition, healthcare providers should be trained to provide comprehensive and inclusive care to transgender individuals, including the identification and management of suicide risk.

## Supplementary Information

**Additional file 1:** PRISMA 2020 Checklist.

## Data Availability

The data extracted for analyses are available from the corresponding author upon reasonable requests.

## Abbreviations

FTM: Female to male
MTF: Male to female
NOS: Newcastle–Ottawa Quality Assessment
WHO: World Health Organization
CINHAL: The Cumulative Index to Nursing and Allied Health Literature
PRISMA: The Preferred Reporting Items for Systematic Reviews and Meta-Analyses
