# Correction: Prevalence of suicidal thoughts and attempts in the transgender population of the world: a systematic review and meta‑analysis

**DOI:** 10.1186/s12991-026-00688-9

**Published:** 2026-07-02

**Authors:** Parisa Kohnepoushi, Maziar Nikouei, Mojtaba Cheraghi, Parsa Hasanabadi, Hamza Rahmani, Maryam Moradi, Ghobad Moradi, Farhad Moradpour, Yousef Moradi

**Affiliations:** 1 Student Research Committee, Kurdistan University of Medical Sciences, Sanandaj, Iran; 2 Social Determinants of Health Research Center, Research Institute for Health Development, Kurdistan University of Medical Sciences, Sanandaj, Iran

**Correction: Annals of General Psychiatry (2023) 22:28**

10.1186/s12991-023-00460-3

In the original publication of this article [1], the author identified the following errors in Table 1. The study by Raymundo et al. (2021) from Brazil was inadvertently listed twice under two different author names (“Raymundo I” and “Chinazzo I”). Both entries refer to the same study and contain identical sample size and outcome data.

The duplicate record had been removed during the final data verification process before the meta-analysis was conducted. Therefore, all quantitative analyses and pooled prevalence estimates were performed using the correct dataset of 71 unique studies. The corrected Table 1 is presented below with the removal of duplicate entry.

**Table 1:** The characteristics of included studies

Author	Year	Country	Study design	Study population	Mean age	Sample size	Suicidal ideation	Suicide attempt	NOS score
Andreas K	2021	European countries	Cross-sectional	TS	30.7	5267	1827	1797	7
Yossi Levi-B	2022	Israel	Cross-sectional	TS	55	195	151	77	6
Shira M	2009	USA	Cross-sectional	FTM	47	153	NR	9	6
				MTF	47	153	NR	12	
Brandon D. L. M	2015	Argentina	Cross-sectional	FTM	30	482	NR	18	6
				MTF	30	482	NR	141	
Robin M. M	2002	USA	Cross-sectional	TS	36.88	73	27	17	7
Azar N	2021	Iran	Cross-sectional	MTF	27.6	127	91	64	7
Arianne R	2021	Brazil	Cross-sectional	MTF	30	763	191	238	7
Sari L. R	2014	USA	Cross-sectional	TS	37.3	31	18	9	7
Katharine A.R	2019	UK	Cross-sectional	FTM	19.7	210	108	46	7
				MTF	20.2	105	58	24	
Ankur S	2021	California	Cross-sectional	TS	NR	120	NR	17	6
				VTS	NR	120	NR	51	
Gareth J. T	2020	Australia	Cross-sectional	TS	26	392	157	66	6
				VTS	26	392	367	209	
Raymond P.T	2017	USA	Cross-sectional	TS	NR	170	62	NR	6
					NR	170	99	NR	
					41.14	36	14	NR	
					41.14	36	19	NR	
Jack L. T	2020	USA	Cross-sectional	TS	23.4	3494	3129	1775	8
Sahika Y	2015	Turkey	Cross-sectional	MTF	NR	42	23	13	7
				FTM	NR	99	55	29	
Galit Z	2018	Sweden	Cross-sectional	MTF	NR	149	60	54	8
				FTM	NR	187	75	79	
				TS	NR	796	283	251	
Sav Z	2021	Australia	Cross-sectional	TS	28	928	NR	394	8
Ashley A	2020	USA	Cross-sectional	TS	NR	372	320	208	7
Greta R. B	2015	Canada	Cross-sectional	TS	32.7	380	133	43	7
Runsen C	2019	China	Cross-sectional	MTF	23.3	687	413	141	7
				FTM	23.3	622	319	68	
Ricardo de M. R. R	2021	Brazil	Cross-sectional	MTF	NR	345	163	64	6
Anthony F	2020	USA	Cross-sectional	TS	NR	572	85	184	6
				VTS	NR	572	40	184	
Peter G	2012	USA	Cross-sectional	TS	37.1	290	NR	83	6
Arnold H. G	2016	USA	Cross-sectional	TS	18	129	52	29	6
Shelley H	2021	USA	Cross-sectional	TS	22.5	3471	977	179	6
		Canada	Cross-sectional	TS	22.5	717	196	54	
Adam G. H	2020	USA	Cross-sectional	MTF	NR	33	10	8	7
				FTM	NR	98	45	30	
Sanna S	2021	Nepal	Cross-sectional	MTF	NR	139	58	26	7
Yiu T. S	2017	Hong Kong	Cross-sectional	TS	NR	106	71	22	6
Seishi T	2011	Japan	Cross-sectional	MTF	32.2	188	141	NR	6
				FTM	26.4	312	219	NR	
Brian C. T	2019	USA	Cross-sectional	TS	NR	1148	974	577	7
Galit Z	2018	Sweden	Cross-sectional	MTF	NR	149	60	54	7
				FTM	NR	187	75	79	
Sav Z	2021	Australia	Cross-sectional	TS	NR	928	NR	399	7
Rao A	2019	Pakistan	Cross-sectional	TS	39	156	67	NR	6
Itala R. C	2019	Brazil	Cross-sectional	TS	26.8	378	256	163	6
Collier M. C	1997	USA	Cross-sectional	MTF	32	318	38	NR	7
				FTM	30	117	25	NR	
Rachel L	2016	Lebanon	Cross-sectional	TS	27	54	NR	25	7
Aboussouan A	2019	USA	Cross-sectional	TS	49.01	154	NR	110	7
				TS	49.01	159	NR	56	
				TS	31.11	2841	NR	1540	
				TS	31.11	1131	NR	226	
				TS	49.01	154	98	21	
				TS	49.01	159	43	6	
				TS	31.11	2841	1936	392	
				TS	31.11	1131	432	40	
Yockey A	2020	USA	Cross-sectional	TS	NR	790	272	287	7
Azeem R	2019	Pakistan	Cross-sectional	TS	39.26	156	67	NR	6
Bauer G	2015	Canada	Cross-sectional	TS	32.7	380	NR	43	7
Boyer L	2021	USA	Cross-sectional	TS	NR	8112	NR	66	7
Chang R	2022	China	Cross-sectional	MTF	34	198	73	NR	7
Chen R	2019	China	Cross-sectional	MTF	22.89	687	413	141	7
				FTM	23.78	622	319	68	
				TS	23.31	1309	732	209	
Chen Y	2020	China	Cross-sectional	MTF	27.9	250	55	64	7
Clements-N. K	2006	USA	Cross-sectional	MTF	NR	392	NR	127	7
				FTM	NR	123	NR	39	
Drakeford L	2018	USA	Cross-sectional	TS	NR	6450	NR	3394	8
Duffy M	2018	USA	Cross-sectional	TS	NR	122	92	91	7
				TS	NR	436	102	33	
Green A	2021	USA	Cross-sectional	TS	19.95	1216	NR	173	7
				TS	16.91	4537	NR	956	
Grossman A	2007	USA	Cross-sectional	MTF	17.5	31	12	6	7
				FTM	19.5	24	13	8	
Jin H	2020	USA	Cross-sectional	MTF	23.2	297	56	NR	7
Kittiteerasack P	2020	Thailand	Cross-sectional	TS	NR	96	22	NR	8
				TS	NR	96	39	12	
Kota K	2020	USA	Cross-sectional	MTF	35	92	30	NR	7
Kuper L	2018	USA	Cross-sectional	TS	21.07	1896	1587	207	7
Lehavot K	2016	USA	Cross-sectional	TS	49.28	212	120	68	7
Maksut J	2020	USA	Cross-sectional	MTF	NR	381	226	50	8
Marshall B	2015	Argentina	Cross-sectional	TS	30	482	NR	159	7
Perez-Brumer A	2017	USA	Cross-sectional	TS	15.23	7653	2467	NR	7
Perez-Brumer A	2015	USA	Cross-sectional	TS	32.74	1229	NR	355	8
				TS	32.74	1229	NR	51	
Peterson C	2016	USA	Cross-sectional	TS	17.1	96	NR	27	6
Rabasco A	2020	USA	Cross-sectional	TS	26.44	133	NR	62	6
Rabasco A	2021	USA	Cross-sectional	TS	26.01	180	NR	75	6
Schweizer V	2020	USA	Cross-sectional	MTF	NR	229	131	NR	7
				FTM	NR	121	94	NR	
Seelman L	2016	USA	Cross-sectional	TS	31.02	2325	NR	1081	6
Mustanski B	2010	USA	Cross-sectional	TS	NR	20	2	2	6

MTF: male to female; FTM: female to male; TS: transgender; VTS: veteran transgender
